# Research in government and academia: the case of health policy

**DOI:** 10.1186/s13584-018-0230-3

**Published:** 2018-10-01

**Authors:** Sherry Glied, Raphael Wittenberg, Avi Israeli

**Affiliations:** 1 Robert F. Wagner Graduate School of Public Service, New York University, New York, USA; 2 Centre for Health Service Economics and Organisation, Nuffield Department of Primary Care Health Sciences, University of Oxford, Oxford, England; 3 Personal Social Services Research Unit, London School of Economics, London, England; 4 Ministry of Health, Hebrew University – Hadassah Medical Center, Jerusalem, Israel

**Keywords:** Knowledge transfer and exchange, Policymakers, Evidence informed policymaking

Making effective health policy requires expert knowledge of an ever-changing technological, epidemiological, social, and economic context. One important vehicle for integrating expert research into the policy process is through linkages and exchanges between researchers and government officials [1]. Many governments respond to this need for expert knowledge by using advisory boards that include academics. Many also integrate academic health policy researchers more fully into policymaking by making them short-term or long-term employees. Each of us (Sherry Glied, Raphael Wittenberg and Avi Israeli) has spent some time as an academic and as a regular member of a governmental policy-making body, in the US, England, and Israel, respectively. In this paper, we reflect on the lessons learned from our dual perspectives.

## Background

There is growing international interest in improving the ability of policymakers to make use of health policy research [2–4] Prior research has examined the effectiveness of varying strategies for knowledge transfer and exchange. These strategies range from dissemination approaches, such as producing succinct summaries of research findings accessible to policy-makers; to training efforts, such as conducting workshops involving researchers and decision-makers; to the use of outside consultants. A considerable literature has examined the structures, processes, and purposes under which research findings are used—or not used-- by policymakers. Across many contexts, countries, and time periods, this literature consistently identifies collaborative relationships as critical to the process [5–10]. Policy intermediaries, with strong ties to both research and policymaking, are important actors in these collaborative relationships [11, 12]. A comprehensive review concluded that “having personal contacts and building trust through quality relationships over time” are key factors in successful knowledge transfer [13]. Most efforts at building such relationships have involved bringing policymakers into the academy. But an alternative approach is to embed academics in policymaking institutions. This article describes how that approach has worked in practice in three contexts.

## Role of health policy academics in government

Although the government structures and health systems of the US, UK, and Israel are quite different, all three make some use of health policy academics in government. In all three countries, academics serve on ad hoc taskforces, standing committees, and other consultative bodies. To varying extents, in all three countries, some senior members of the permanent civil service, particularly in the scientific and statistical agencies, come from academia and, in some cases (often in Israel), may maintain their academic roles while serving in government. For example, the last four Chief Statisticians in Israel have been academics, and two recent Chief Analysts at the Department of Health and Social Care for England (DHSC) had previously been academics. For reasons that we discuss below, this flow is usually one way – from academia into government – though there are occasional exceptions. Academics who move into the permanent civil service often acquire a professional identity as government officials and rarely return to academia.

In all three countries, academics are often invited to act, on a short-term, temporary basis, as advisers or members of specialist government committees. In England, parliamentary Committees, such as the House of Commons Health Committee, also appoint academics as advisors on specific enquiries. In the US, scientific advisory boards that make critical policy decisions, such as the United States Preventive Services Taskforce, are often staffed largely by academics. In Israel, academics often form part of the committees that update the medical basket. In these situations, the inclusion of academics is a tactic that may serve to remove a decision that depends on evidence from the partisan political process [14]. Academics serving on taskforces rarely migrate into government – they retain their professional identities as academics.

Finally, in all three countries, there are situations where policy researchers hold full-time (though often temporary) positions within the bureaucracy and retain a connection to academia, for example, by taking leave from a position to which they intend to return. The specific parameters of these situations vary among countries.

In England, academics may be seconded for a limited period to government departments. Some academics, often clinician experts, work for the Government, with contracts for a specified time period, before returning to academia. This pattern is most common among specialists such as analysts, research managers and clinicians at senior and mid-level. Conversely civil servants may be seconded to university research units. Wittenberg was for many years seconded part-time from the Department of Health and Social Care to the London School of Economics and Political Science (LSE) before he became a member of LSE and University of Oxford staff; and a few of his LSE colleagues have been seconded for periods to the Department of Health and Social Care or other government departments. In some circumstances, academics in England simultaneously serve in government and maintain their positions in academia.

In the United States, academics typically hold temporary full-time positions in government. Many universities limit the length of time that an academic may spend on leave in such a position (a practice known as the Kissinger rule) [15]. While some academics in government may continue to teach as adjunct or part-time faculty, it is unusual for someone to hold both a full-time academic position and a position within government (other than as a part-time member of an advisory board or review committee) at the same time.

In Israel, health policy academics frequently serve as members of government-appointed committees, or as advisors to senior government officials. It is rare to see a person who has a full-time position in academia move to a full position in the Ministry of Health, either permanently or temporarily. However, in Israel it is possible for professionals to combine academic and civil service pursuits without having to sacrifice one for the other. The most common arrangement is for a senior medical professional to hold a long-term, full-time position in government along with a part-time connection with a university. Physical proximity and the tight knit networks linking professionals in the two settings encourage such arrangements. In some cases, the part-time connections with a university involve an opportunity to move up the academic ladder (including up to full professorial rank), based on research and publications and other academic achievements. The academic affiliation, worn with a sense of prestige, reflects the interest and involvement of government officials in academic work, and the value they assign to it.

Avi Israeli has served full-time as Director General of the Ministry of Health (2003–2009), currently serves part-time as Chief Scientist of the Ministry of Health, and has served as Chair of the Public Committee to Update the Basket of Health Services in a voluntary capacity. He served in all three of these governmental roles while also serving as Head of the Department of Health Policy and Management of the Hebrew University-Hadassah School of Public Health. The intensity of his academic involvement varied according to the intensity of his governmental role.

In the US, health policy academics are typically recruited into the executive branch of government to work full-time for a few years (often coinciding with a Presidential term), with the expectation that after term of service they will return to academia. Academics fill a significant share of the politically-appointed positions in the U.S. Department of Health and Human Services, as did Glied, who took a leave from a tenured position on the faculty of Columbia University to serve as Assistant Secretary for Planning and Evaluation from 2010 to 2012.

In this one foot in each camp situation, researchers maintain professional identities as both government officials and academics. This can be complicated. Health policy questions often turn on issues of values with respect to both ends (what is the appropriate distribution of resources?) and ends (how should considerations of collective responsibility and individual liberty be reconciled?). Academics in government inevitably bring their own values to a table crowded with the values of their fellow policymakers. As discussed below, this poses a challenge of balancing academic freedom and commitment to evidence with policy relevance and the political context -- not always a straightforward matter.

## Roles of government in health policy research and analysis

Governments in all three countries play substantial roles in health services and policy research. Governments often conduct major surveys and data collection efforts. Government research funding agencies, including the National Institute for Health Research in the UK, the National Institutes of Health and the Agency for Healthcare Research and Quality (AHRQ) in the US, and the National Institute for Health Policy Research and Center for Disease Control in Israel, fund both investigator-initiated research and research in response to specific programs or calls. Where the government is directly involved in the delivery of health care services, it may also fund a program of health services research aimed at improving the quality of delivery in government-operated facilities. For example, in the US, the Veterans’ Administration funds a very extensive intramural and external program of health services research, though this is not generally focused on broad federal health policy (https://www.research.va.gov/services/hsrd.cfm). Academics may serve on review panels for such research. Academics who have migrated to the permanent civil service may play important roles in developing specific programs or calls.

In the US, Congress has often shied away from using this type of research funding to support policy-oriented research. The AHRQ is an eloquent symbol of that reluctance – it had originally been called the Agency for Health Care Policy Research, but was renamed in 1999 to take the word Policy out of its title [16, 17]. The Department of Health and Social Care for England, by contrast, has a Policy Research Program (PRP) which commissions high quality research based evidence, some of it at a number of PRP research units that undertake substantial long-term programs of research agreed with DHSC. In these cases, governments sponsor academic research, which is undertaken by academics within universities. The resulting reports and publications are usually circulated publicly and may be quoted in Government Green and White Papers. In Israel, the Ministry of Health and the National Institute for Health Policy Research, a mechanism created by the National Health Insurance Law to fund evaluation of the health system, make grants specifically to support policy development.

In addition to promoting academic research, governments also undertake, in house and via contract, non-academic analyses that inform policy or practice in health and social care, and address very specific policy issues. These analyses rarely involve sustained detailed investigation of a specific research question or hypothesis and rarely involve systematic reviews, interviews or other primary data collection, extensive secondary analyses of existing data, or preparation of articles for academic journals. The Department of Health and Social Care in England conducts in-house a wide range of statistical, economic, operational research and other forms of analyses and modelling (as does the Department for Work and Pensions) [18]. These frequently draw on academic research, as well as official data, surveys and other sources of evidence, including research commissioned by the Department of Health and Social Care. In the US, the Office of the Assistant Secretary for Planning and Evaluation (ASPE) and other policy units conduct similar in-house analyses and commission studies through detailed contracts that specify both the question and the nature of the work product. In the US, almost all of this research is conducted by contract research firms (examples include the RAND Corporation, Mathematica Policy Research, MDRC, and RTI). In Israel, the Ministry of Health commissions outside studies. on specific topics and also utilizes independent research organizations such as Myers Joint Brookdale Institute and the Gertner Institute for Health Services Research.

Such government-commissioned research can pose political risks for the commissioning agencies. The research studies sometimes provide results that are not clear cut, are counter-intuitive, do not support the preferred policies of decision makers who posed the questions, or raise new issues, despite being aimed at very specific problems. Academics in government may be particularly useful in seizing opportunities that the results of such commissioned research studies offer, whether by defining follow-up research questions, encouraging redirection of existing policy, or translating research findings into specific policy actions. For example, in 2013, ASPE commissioned research to assess whether the requirements of the 2018 Paul Wellstone and Pete Domenici Mental Health Parity and Addiction Equity Act were being met [19]. The report generally found that substantial gains had been made in the areas of quantitative treatment limits that were the focus of the study. This surprised advocates, who reported instances where parity requirements were not met. The presence of an academic mental health policy expert, Richard Frank, enabled ASPE to commission a new research study focused on the discrepancy [20]. The later study helped shape the Department’s subsequent enforcement activities.

## Structure of research in government and in academia

Successful academic health policy researchers have specialized expertise and work hard – traits that are prized in both academia and government. But while these high level characteristics translate easily, many aspects of the institutional structure of policy research in government and academia are quite different (and these, in turn, differ across countries). Both the kinds of research that are demanded and the ways that work is conducted vary across these two contexts.

The overarching goal of research in academia is to better understand the world. The most prized work provides a new perspective or insight into existing phenomena. Novel, creative work will identify a previously unrecognized problem, propose a new set of methodological tools, or offer a distinctive theoretical structure that ties together ideas or events that had been seen as unrelated [21–23]. This kind of work spawns a flood of follow-on research, normal science, filling in the details of a model. The corresponding metric of success in academia is articles in high quality academic journals and citation counts – measures of how many other researchers found that this work gave them a new and useful way of engaging with problems.

The merit of scholarly work depends critically on its creativity, novelty, and insight. Rigor and accuracy do also matter, but are not sufficient. Most of the onus for ensuring the accuracy of papers lies with the researcher. The external peer review system, in its design, can provide only a top-level review of the researcher’s approach, such as the design of the study, and pose a few questions. Reviewers rarely examine underlying data and often have inadequate time or training to assess the validity of findings [24]. While researchers regularly call for replications and re-analyses, publications and promotions go to those who break new ground. The conventions of the academy also define what a high quality paper should cover. The data, methods, and results sections of a paper must adhere to certain discipline-specific standards, but authors have nearly free reign in choosing their questions and in discussing the implications of their results.

The growth of research funding for the social sciences, and of the field of public policy as an academic pursuit, have narrowed the gap between the goals of researchers in the academy (at least in this field) and those in government somewhat. Government funders of social science research expect investigators to address questions of policy significance and to make an effort to translate their findings into actionable suggestions (this is particularly true in England). Schools of public policy likewise encourage their faculty to study issues that policymakers will find relevant. Nonetheless, this gap remains an enduring concern, as evidenced by a steady flow of books, articles, and conferences and renewed attention to “knowledge transfer” between academia and policymakers [1].

While academic policy researchers do conduct research that may be useful to policymakers, the principal goal of research in government remains quite different from that of the academy-- to give policymakers information that will help them to solve a specific, pre-defined policy problem in real time. In contrast to the broad range of questions an academic might choose, most of the time a researcher in government is given a specific assignment to complete within a specific, often challenging, deadline. While a research team in academia generally conducts research itself, sometimes in collaboration with other research teams, in government most research (except rapid turnaround requests) is not conducted in-house but commissioned under contracts with external researchers . For example, at the Department of Health and Human Services, ASPE was asked to assist the Secretary in developing the benefit package that would be the standard for coverage under the Affordable Care Act. The agency conducted analyses in-house (comparing benefits offered in various markets, for example [25]) and used commissioned research (assessing actuarial values of various benefit designs) that would help answer that very concrete, policy relevant question [26].

For most questions, the permanent civil service staff is more likely to understand how much effort a question deserves, what the realistic bounds of the policy debate are likely to be, and how formally it needs to be answered. On more than a few occasions, the “old hands” in government make clear to time-limited academic appointees that internal bureaucratic politics, data limitations, or the likely uncertainty of any answer argue against devoting a lot of time or budget to what seemed to be an interesting and important policy question. But the academic’s training and socialization to seek creative solutions to new and difficult questions can be valuable when a novel policy problem is far from the questions that “old hands” have dealt with in the past.

The questions that flow to academics in government are usually very tough, out-of-the-box challenges that can best be answered by bringing together the worlds of institutional knowledge within government and of creative research in academia. Collaborations between governments and academics, often with academic researchers in government serving in a bridging role, can take the form of extended programs of policy-oriented research.

Such programs have generated useful results that altered the shape of policy. For example, the analysis prepared for Ministers in successive UK governments and expert enquiries on reforming the system for financing long-term care involved close collaboration between researchers at the Personal Social Services Research Unit at the London School of Economics and analysts in the social care analytical unit at the DH [27, 28]. (Wittenberg was for many years a member of both). The development and implementation of the Prospective Payment System (DRGs) in the US involved a decades-long collaboration between the Office of Research and Development in the Health Care Financing Administration and academics at several Universities [29–31]. In Israel, a group of Israeli academics began to study the issue of evaluating, measuring and reporting on the quality of community based health care. With time, these researchers established connections with health plans, in which they discussed the literature on quality measurement, compared different measures used in the different plans, and committed themselves to deploy agreed measures to improve quality of care, while committing not to reveal measures of quality in the different health plans. In 2004, Israeli, as The Director General of the Ministry of Health, an academic serving in government, and aware of this research, offered to place the program under the aegis of the MOH, along with full funding through the National Institute for Health Policy, leading to this academic-initiated activity transforming into the National Program for Quality Measurement in Community Care. It combines research and comparisons with other countries, a framework for health plans and providers to continually improve quality of care, and reporting of quality measures to the public.

As these examples suggest, while the primary intention of government-commissioned research is to address specific policy issues, not to generate academic publications of generalizable value, sometimes the two are quite compatible. In the field of health policy research, the most notable example of such a happy symbiosis is the RAND Health Insurance Experiment. In 1971, the US Department of Health, Education, and Welfare, which was then considering options for universal health insurance, funded the RAND Corporation (a US contract research firm) to was commissioned to produce estimates of the effects of alternative health insurance packages on service utilization. The results of the study remain of direct policy significance; the study also generated hundreds of peer-reviewed journal articles which have spurred further academic research [32, 33].

While the academic perspective may be useful in identifying a path to an answer for a challenging question, the academic’s usual approach is unlikely to be helpful in government. In contrast to the academy’s preference for novelty, research in government is most useful when it is straightforward; uses well-accepted, conventional methods; and can be replicated easily. Meta-analyses and reviews of existing literature are preferred over original research. In government, rigor and accuracy trump imagination and cleverness every time. In contrast to the academic model under which a small number (2–4) of voluntary peer reviewers assess the validity and interest of a completed work and pose a few questions, a major piece of governmental policy research, one that will be released publicly, may go through extensive internal review. Junior researchers within government may check the math; outside peer reviewers may be enlisted – and paid – to ensure that methods have been used appropriately; and legal staff, political staff, even public relations staff will review the product from their various perspectives. The framing of the question and the discussion section will garner at least as much scrutiny as the methods. The difference in processes means that at least according to formal requirements, a policy research report released by a government is often more likely to contain results that are accurate, verifiable, nuanced and replicable than a paper published in the most esteemed peer-reviewed journal (see https://aspe.hhs.gov/report/hhs-guidelines-ensuring-and-maximizing-quality-objectivity-utility-and-integrity-information-disseminated-public). Research on the quality of government-commissioned research in England finds that it is likely to be more accurate than other commissioned research, though it may not be published in a timely fashion [34, 35]. It is also likely to be less broadly interesting, provocative, and creative than academic research. It can be tremendously valuable if it is used by policymakers in shaping policy – or in rejecting a contemplated policy direction -- even if it is never ever cited again.

These differences in the nature of publication help explain why excellent policy analysts in the permanent civil service rarely migrate back into academia. Their commitment to doing the kind of research needed by government, and to publishing through the government process, often means giving up the chance to pursue their own research interests and to publish extensively in academic journals. Even the most well-established and effective policy researcher in government is very unlikely to have a resume of novel academic publications that would satisfy a university hiring committee. Moreover, if the researcher has become identified with specific policy directions (whether or not based on his or her research), and those policy directions are contested, return to academia may be more unlikely. While efforts are underway to facilitate movement back into academia, for example by encouraging tenure review committees to consider a broader range of publications (see, for example, http://wtgrantfoundation.org/grants/institutional-challenge-grant; http://www.cahs-acss.ca/wp-content/uploads/2011/09/ROI_FullReport.pdf), this difference in the nature and number of work products and tasks explains why it is so often challenging to move from full-time government service into academia.

## Consuming policy research in government

The interplay between research and policy is strongly molded by politics [36, 37]. Within this political context, policymakers make use of research evidence in many ways, from informing a policy decision to justifying or legitimating one to compensating for the lack of policy action [14, 38]. This multiplicity of uses makes the connections between research and policymaking are often indirect and obscure [6, 14, 39]. This is particularly true in health policy, where policymakers must often balance considerations of efficiency with their own and their constituents’ values about the appropriate distribution of resources. A growing consensus, however, suggests that research, broadly defined, affects policymaking across many dimensions. Whether the use of research evidence actually improves the quality of government services, and whether it enhances democratic decisionmaking, remain contested questions [37, 40, 41].

Academics in government are useful because they can conduct research – but they are also useful in other ways. Full-time policy researchers in government do not routinely read academic research. There are several reasons for this lack of attention. First, the timescale of academic research is often not appropriate to government [1]. Most academic research is focused on understanding the world as it is now – but most policy researchers in government need answers to specific questions to inform decisions about particular future policy choices. Academic research happens at its own pace (unless commissioned by government agencies), but governments make decisions when they must or can, often on a very short time horizon. Second, academic research products are not necessarily organized to be useful to policymakers. A journal issue, with its assortment of articles, may contain nothing of interest to a policymaker whose needs are very specific. Even a single article likely contains considerable spurious information (from the policymaker’s perspective) but lacks some specific detail (how would this intervention affect a particular population?). Academics in government can connect the findings of academic researchers and the needs of policymakers: this can be a crucial part of their role, in view of their expertise in both research and in policy analysis.

Academic researchers make this connection in a variety of ways. Their knowledge of the academic research literature allows them to identify opportunities to insert existing research into policy discussions. This insider knowledge allows them, where appropriate, to provide research evidence to political champions willing to support evidence-based policies, or to help politicians develop evidence-based agendas in support of policy goals. Academic researchers can also strengthen the value of government commissioned research, by situating it in the context of the broader literature. The success of academic researchers in achieving these ends will depend on their legitimacy within the research community and their institutional positions within the policymaking body [42] Academics are also more likely to be successful if they understand the constraints and demands of both the scientific and policymaking communities [43].

In playing these roles, academic researchers in government come to appreciate that research results are only one of many factors that influence policy design. In government, the academic finds that no research result stands on its own – as it would in the context of academic peer review. Instead, research enters the process through a complex network of politicians, decision makers, policy agendas, media coverage and pressures from interest groups (sometimes citing academic research) and the general public. Effective academics in government can put their creativity and knowledge to use here as well. They can help put research in context, compare conflicting findings, connect research to ongoing agendas, link research to powerful allies, and provide rationales for policy support. While research may not always carry the day, academics in government are well placed to inject research findings into debates where they have not found a place before. At the same time, the enlistment of researchers to support certain positions preferred by policy makers must be taken into account: creation of mechanisms, such as symposia of government based scientists, can balance competing positions supported by research.

## Conclusions

The interplay between academia and government, which exists in all three countries, has strengthened the quality of health policy. It has also improved the quality of academic research in this area. Health policy is ultimately bound by institutional, political, and financial constraints and health policy research is both most valuable and also most interesting when it recognizes and responds to those constraints. This need not mean that academics must give up their freedom to choose topics and pursue creative ideas. On the contrary – the ability to go beyond the specific problem at hand is where the academic setting is most valuable. But academic research is itself more robust, and more useful, when it recognizes the institutional constraints of legislation and implementation.

Academic research will translate more directly into policy when it makes sense within a specific policy context. It will also have more impact if it is readily brought to the attention of policymakers. Academics can help make that happen by collaborating in the production of Briefs and Research Summaries, often by working with think tanks (particularly those that are less ideological) and similar bridging organizations. These products are far more likely to be read, and so have a greater chance of entering the policy process, than academic papers. Even more important, academics can bring their research to policy attention by seeking out opportunities to talk to and collaborate with policymakers. That requires time, and is sometimes a distraction from the task of publishing peer-reviewed journal articles, but such collaborations offer not only policy impact, but often promising areas of research, access to useful data, and wise advice from experts in the field. Finally, academics can profit from spending time in government, where they can both learn about this alternative culture and build lasting relationships that will help translate their future research into policy [44, 45].

Making research meaningful to policymakers is increasingly key to professional success. In the UK, the Research Excellence Framework, the official system for assessing the quality of research in UK higher education institutions, takes account of the impact of research as well as its scientific quality. Government-initiated and researcher-initiated health policy research both need to have policy relevance and impact as well as high scientific quality. While the US has no parallel mechanism, research funders, including both public funders and private foundations, increasingly insist that researchers document the impact of their research on policy and well-being. In Israel, research funders often emphasize the importance of policy relevance and dedicated journals, such as the IJHPR, help to raise the academic profile of policy related research.

Playing a role in the policy process is not only professionally valuable, it is also immensely personally rewarding (As each of us can attest). Academics often become health policy researchers because they care deeply about their nations’ health systems, and it is very satisfying to make meaningful contributions to their success. As scholars, service in government offers an unparalleled opportunity to discover a new set of problems and audiences. As individuals, working in government allows scholars to encounter a new set of colleagues and friends, with different perspectives and interests. Weaving research, often based on access to unparalled sources of data stored by government (and not readily available to university based academics), into policy discussions and thus experiencing its actual impact on policy outcomes for the country’s population is hugely satisfying to the academic functioning in government.
